# Ascertaining injury risk issues through big data analysis: text-mining based analysis of national emergency response data

**DOI:** 10.3389/fpubh.2024.1326457

**Published:** 2024-02-28

**Authors:** Jin-Young Won, Yu-Rim Lee, Myeong-Heum Cho, Yun-Tae Kim, Ji-Hyang Lee

**Affiliations:** ^1^National Disaster Management Research Institute, Ulsan, Republic of Korea; ^2^National Fire Research Institute of Korea, Asan, Republic of Korea

**Keywords:** injury, text-mining, EMS data, novelty, scalability, health policy

## Abstract

**Objectives:**

Injury prevention can be achieved through various interventions, but it faces challenges due to its comprehensive nature and susceptibility to external environmental factors, making it difficult to detect risk signals. Moreover, the reliance on standardized systems leads to the construction and statistical analysis of numerous injury surveillance data, resulting in significant temporal delays before being utilized in policy formulation. This study was conducted to quickly identify substantive injury risk problems by employing text mining analysis on national emergency response data, which have been underutilized so far.

**Methods:**

With emerging issue and topic analyses, commonly used in science and technology, we detected problematic situations and signs by deriving injury keywords and analyzing time-series changes.

**Results:**

In total, 65 injury keywords were identified, categorized into hazardous, noteworthy, and diffusion accidents. Semantic network analysis on hazardous accident terms refined the injury risk issues.

**Conclusion:**

An increased risk of winter epidemic fractures due to extreme weather, self-harm due to depression (especially drug overdose and self-mutilation), and falls was observed in older adults. Thus, establishing effective injury prevention strategies through inter-ministerial and interagency cooperation is necessary.

## Introduction

Injury is defined as “a harmful outcome in terms of physical and mental health that occurs as a result of an intentional or unintentional accident” and constitutes a leading cause of subsequent disability and death worldwide (1). Injuries are preventable and hold significant importance for public health. In 2001, the World Health Organization recommended the establishment of health-centered national injury surveillance systems to enable a scientific approach to injury prevention. Different countries have established surveillance systems, which are categorized based on the injury severity and the use of passive or active surveillance. In the United States, the National Hospital Discharge Survey at the inpatient level, alongside the National Electronic Injury Surveillance System-All Injury Program and the National Hospital Ambulatory Medical Care Survey at the emergency department level, constitutes such a system. Australia uses the hospital-based National Hospital Morbidity Database as a data source for operating injury surveillance systems with separate categorizations for injury cases. Canada operates the National Trauma Registry at the inpatient level, in addition to the National Ambulatory Care Reporting System and the Canadian Hospitals Injury Reporting and Prevention Program (CHIRPP) at the emergency department level. Since 2005, South Korea has established a national integrated injury surveillance system and has gradually introduced a medical institution-based injury surveillance system. In 2006, using the emergency department medical system, the emergency department-based injury in-depth surveillance was introduced to assimilate injury data. To overcome limitations arising from the production and management of injury surveillance data by various relevant ministries based on the place, object, and activity of the injury, national injury comprehensive statistics have been published since 2010. This effort aims to integrate and standardize injury-related data that are generated in various forms to ensure comparability among the data and to identify the scale and characteristics of injuries in South Korea (2). The national injury statistics, along with the detailed injury surveillance data that they comprise, are utilized to identify injury issues in each area and to establish and implement preventive policies.

One of the most important elements of an injury prevention policy is the “mechanism,” which refers to how the injury occurred and how the person got hurt, which be seen as a crucial area within injury policy issues. In policy science, a policy problem is an unrealized value or opportunity for improvement, and information about the nature, scope, and severity of the problem is obtained by applying a problem-structuring process. Problem structuring constitutes the steps of problem search, problem delineation, problem specification, and problem sensing, and is initiated by detecting early signs of widespread worry and stress (3). Furthermore, a public health approach to injury prevention starts with problem identification and surveillance procedures to collect and analyze data, thereby structuring the severity of the problem and its targets (4).

Injury has a broader, more comprehensive scope than an abnormality or disease (5). Additionally, injury risk factors and vulnerable groups are constantly changing due to external environmental factors, such as climate change, demographic and social structural changes, and technological advancements. The components, causes, and consequences of injury problems are relatively broad and complex as compared to other areas, which makes it challenging to identify actual injury problems and establish preventive policies. There are two main limitations in the extant injury surveillance and utilization systems with regard to the timely identification of real problems across a wide range of injury domains.

First, there is a limitation in the data. As most injury surveillance data are entered according to a standardized registration system to reduce errors in the input process and efficiently perform quality-control processes, there is a possibility that detailed information on accident-site conditions and injury causes may be missing. Moreover, there is a significant time delay in utilizing the data due to the processing and management of very large amounts of data. The national emergency response data comprises recordings made by paramedics on the details of their activities in the emergency medical service (EMS) activity logbook in accordance with Article 18 (Maintenance of Records of Emergency Medical Service Activities) of the Enforcement Rules of the Act on 119 Rescue and Emergency Medical Services. Particularly, EMS activity logbook describes the paramedic’s assessment of a severe trauma patient according to the prescribed format and additionally records the associated circumstances and witness statements as necessary. As these data are collected in real time, quick checking of the raw data through the system and the utilization of the data as one of the sources of national injury statistics are possible. Some of the data limitations can be overcome by utilizing national first-responder data.

Second, research and policies often focus on microscopic injury issues in specific areas, which had led to a lack of research for the identification of macroscopic injury issues across all areas of injury. Existing studies related to injury prevention policies either identify influencing factors and risk factors through empirical studies by using diverse injury surveillance data or analyze the occurrence trends and characteristics of standardized injury accident types and risk factors. For instance, mortality data from the United States Centers for Disease Control and Prevention were used to calculate fall mortality rates by sex, age, race, ethnicity, and residency status (6), and mortality surveillance data from the Disease Surveillance Points (DSP) system in selected areas of Guangdong Province, People’s Republic of China, were used to identify priorities for government intervention based on the cause-of-death code (7). In Canada, the migration of the emergency department-based injury and poisoning surveillance system from a centralized data entry process (CHIRPP) to an online distributed process (eCHIRPP) revealed that unintentional injuries were the leading cause of death of Canadians aged 1–44 years (8). Moreover, studies using hospital inpatient discharge record data from the Wisconsin Bureau of Health Information found that alcohol-related problems and mechanical and motor problems significantly increased the risk of a diagnosed fall among inpatients aged 65 years and older (9). In San Francisco, trauma registry data, medical records, and outpatient mental health care data from the Billing Information System of the Department of Public Health showed that 20% of patients who were hospitalized for unintentional injuries were diagnosed with a mental illness (10). Although some studies have explored effective linkages between ambulance records and hospital records (ER, discharge) (11–13), none have addressed the issue of macroscopic injury through an evaluation of ambulance records. In Australia, a mental health and self-harm module was developed using paramedic electronic patient care data derived from the National Ambulance Surveillance System (14).

With the rapid development of information and communication technology and digital services, data-driven decision-making has become a priority in the policy-making process. In recent years, the importance of text mining to support policy has been increasingly recognized by various organizations (15). In the field of science and technology, which has a relatively large amount of refined data, new objective studies are being promoted to identify emerging issues by utilizing text mining from a policy-making perspective. Topics from patents have been extracted to identify promising and unexploited technological areas for wireless power transmission through topic clustering, time-series analysis, and the application of technology innovation cycles (16). A model to detect emerging trends was developed by associating features extracted from scientific articles with each topic and ranking them based on interest and usefulness (17). Other studies have proposed group dynamics approaches to analyze issue trends, explored continuous changes in knowledge cluster keywords to detect emerging issues with new directional changes (18, 19), identified various connections between issues through correlations between node characteristics in the network (20), and recognized the threats and opportunities that are posed by issues with automated programs (21). Furthermore, several commercial systems have been introduced to detect emerging trends in textual data through factors such as linguistic and statistical features, learning algorithms, training and test-set generation, visualization, and evaluation (22). With the setting of criteria by utilizing various metrics to identify emerging issues and topics and conducting evaluation tests, the following attributes need to be met: radical newness, coherence of the topic, relatively rapid growth, and scientific impact of the topic (23–25).

We proposed a method for discovering a large number of injury problem representations and deriving a substantive problem. This study was conducted with an aim to ascertain a novel method to explore injury risk issues and identify injury problems by applying the concept and analysis methodology of emerging issues, which is actively used in the field of science and technology. We tested this proposed method by using national emergency response data, where the situation of the accident site and injury background have been recorded in detail. Among the four attributes of the emerging issues that have been identified in the literature, topic coherence and scientific impact are useful criteria for exploring topics of interest in science and technology; however, these criteria are somewhat heterogeneous to apply in the injury domain. Thus, in this study, injury risk keywords were selected based on radical newness (novelty) and rapid growth (scalability), and various connections between keywords were identified through semantic network analysis.

## Methods

### Research framework

**Figure 1:**
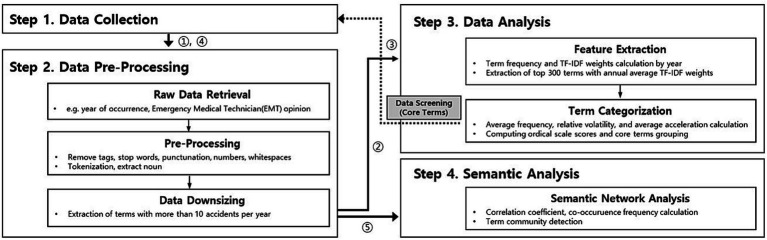
Analysis flowchart.

This study was broadly divided into data collection, data pre-processing, data analysis, and semantic analysis (Figure 1). The RcppMeCab (Kim and Kudo), igraph (Csardi), tidytext (Silge and Robinson), tidyvers (Wickham), tidygraph (Pedersen), ggraph (Pedersen), and widyr (Robinson) packages in R were used for text mining.

### Data collection

This study is based on the most recent 10-year first-aid data from 2013 to 2022, and the research site is confined to Chungcheongnam-do because of the study site’s status as a mixed urban–rural area. In this region, cities with concentrated economic power and infrastructure, adjacent to the metropolitan area, coexist with rural areas where agricultural and marine products are cultivated. This coexistence creates a diverse range of injury accidents, which are anticipated as a result of rapid social changes. The emergency response data are derived from the rescue activity logs of 16 fire departments and 80 safety centers, excluding the Chungcheongnam-do Fire Headquarters and the 119 Special Response Team. The selection of injury accident data is based on “transport classification,” “non-illness accident type,” and “evaluation findings” from the overall emergency response data. Specifically, only cases where the patient is transported by ambulance due to injuries sustained in a non-medical accident, such as a fall, poisoning, or laceration, were included in the analysis. To enhance the efficiency of the analysis, traffic accident data (involving drivers, passengers, and pedestrians) were excluded. This exclusion was justified by the relatively clear mechanism of injury for traffic accidents, wherein the cause has been systematically investigated and analyzed. The evaluation findings encompass various aspects, including the report, patient demographics, location and symptoms, patient assessment details, and on-site emergency care and medical guidance.

### Data pre-processing

#### Pre-processing

Pre-processing involves the initial processing of text data for analytical purposes. A corpus of “evaluation findings” items was constructed from the raw data for analysis, and the corpus underwent tokenization to eliminate noisy data. A “token” is a grammatically indivisible unit, and “tokenization” involves separating tokens from a corpus. Depending on the method and purpose of the analysis, tokenization criteria can include whitespace, morphemes, and nouns; this study focused on morphemes. Although English sentences comprise a typically straightforward arrays of words, Korean (Hangul) words often consist of more than one morpheme, and many words are used inertly and without meaning. For the sake of analysis efficiency, only nouns, which carry the most information in a sentence and can help identify the injury mechanism, were extracted from the nine parts of speech (noun, pronoun, rhetoric, investigation, verb, adjectives, adverbs, articles, and interjections) in Korean. However, during the translation of the results of the analysis into English, a term may sometimes be expressed as a phrase.

#### Data downsizing

The data were transformed into a term-document matrix data frame format. Documentation refers to the paramedic’s assessment of each injury accident. The average number of injury accidents is 25,073 per year, and the total number of noun terms extracted from the corpus averages 7,757 per year. To streamline the data and enhance analysis efficiency, only terms with a Document Frequency (DF) of 10 or higher, indicating the number of accidents in which a particular term appears, were selected. According to Bird and Loftus’ Safety Management Approach, 630 non-injury accidents provide many opportunities to prevent 10 minor accidents and one major injury (26). This implies that accidents involving 10 or more injuries in a given term per year serve as precursors to major injury accidents, but not isolated events. Reducing the data to terms with a DF of 10 or higher results in an average of 1,862 terms per year; moreover, after eliminating duplicate terms, a total of 2,961 terms were included in the final term list.

### Data analysis

#### Term importance analysis

Term frequency (TF) is the number of occurrences of a term (t) in an individual document (d) divided by the total number of terms in that document. It emphasizes terms that appear more frequently within a document as being more crucial for describing the document’s content. However, TF alone may not always perform optimally, leading to the introduction of inverse document frequency (IDF) to address this limitation. IDF decreases as a term is mentioned more often across the entire corpus and increases as it appears less frequently. Essentially, IDF quantifies the specificity of a term as an inverse function of the number of documents in which it appears (27). TF-IDF is the product of TF and IDF, incorporating a weighting mechanism that is directly proportional to term frequency and inversely proportional to document frequency (Equation 1). TF-IDF, a widely used heuristic method in information retrieval, increases the weight of terms occurring frequently in a document (Equation 2) and decreases the weight of terms occurring frequently across documents (Equation 3) (16). As different documents may have distinct TF values for the same term, the maximum TF value per term is used to calculate the TF-IDF weight. The top 300 terms are selected as candidate injury keywords based on TF-IDF weights.


(1) $$TF-IDF\;\left(t\right)=TF\left(t\right)\times IDF\left(t\right)$$

(2) $$TF\left(t\right)=\frac{\mathrm{number}\;\mathrm{of}\;\mathrm{times}\;\mathrm{term}\;t\;\mathrm{apprears}\;\mathrm{in}\;a\;\mathrm{document}}{\mathrm{total}\;\mathrm{number}\;\mathrm{of}\;\mathrm{terms}\;\mathrm{in}\;\mathrm{the}\;\mathrm{document}}$$

(3) $$IDF\left(t\right)=\ln\;\left(\frac{\mathrm{total}\;\mathrm{number}\;\mathrm{of}\;\mathrm{documents}}{\mathrm{number}\;\mathrm{of}\;\mathrm{documents}\;\mathrm{containing}\;\mathrm{term}\;t}\right)$$

### Categorization

In the stage of detecting problem situations and identifying signs of change, the methodology of exploring emerging technologies in the field of science and technology is utilized. Much criticism has been directed at existing research related to emerging technologies for not being suitable for identifying new topics, and to address this, precise definitions and criteria indicators related to emerging technologies have been proposed (23). Utilizing these concepts, in Korea, novelty, fast growth, and cross-sector impact are defined as criteria indicators for emerging issues, and empirical studies have been conducted to swiftly identify comprehensive emerging issue candidates from literature databases (Web of Science) or online news sources (28, 29). In these studies, concerning keyword categorization, the average frequency, acceleration, and relative volatility of each technological keyword were measured, and based on the distribution of each indicator, they were classified into four groups: emerging technology, variable technology, diffusing technology, and undiffused technology. “Emerging technology” refers to rapidly diffusing technologies, “variable technology” refers to technologies that diffuse quickly but have high volatility, “diffusing technology” refers to technologies that have already diffused and matured, and “undiffused technology” refers to technologies that have not yet diffused. The analysis results are being utilized to understand the landscape of Korea’s technological and industrial ecosystem (29).

Building upon this framework of previous studies, this research aims to classify terms based on statistical characteristics according to novelty and fast growth criteria and to define and characterize these features. Novelty is assessed using average frequency and relative volatility, while considering both the occurrence frequency of a term and its temporal variability. Relative volatility represents the relative value of the standard deviation of the frequency of each term over a period. Calculations are performed based on the annual average frequency to gage the extent to which the term’s frequency of occurrence is dispersed each year, indicating rapid changes compared to the average. To account for the increase in the number of terms mentioned over time, the ratio of the standard deviation of a term to the average of the standard deviations of all terms is calculated, as depicted in Table 1.

**Table 1:** Results of the number of EMS activities and terms extracted by year.

	2013	2014	2015	2016	2017	2018	2019	2020	2021	2022	Mean
Casualty incidents	23,916	26,816	26,683	27,323	25,353	25,227	24,894	22,504	23,039	24,975	25,073
Total terms	6,324	6,667	7,019	7,216	7,430	8,085	8,548	8,396	8,747	9,142	7,757
Downsized terms	1,300	1,422	1,486	1,582	1,733	2,025	2,180	2,188	2,271	2,431	1,862

EMS, emergency medical service.

For scalability, the average acceleration is computed to understand the likelihood of continued scaling in the future. The concept of acceleration introduces an incremental acceleration value for the frequency of occurrence of a term each year. This value is accumulated until the year of the last appearance to finally calculate the acceleration value (30).

For each of the 300 candidate injury keywords, average frequency, relative volatility, and average acceleration are computed to rank them, assigning a score ranging from 1 to 5. Based on the score distribution of the three indicators, each term is classified as a “hazardous accident,” “noteworthy accident,” or “diffusion accident” to define its characteristics.

### Semantic analysis

This step involves observing the meaning and impact of injury keywords. To delve into the issue of “hazardous accident” terms with high policy importance, injury accident data containing “hazardous accident” terms were selected for another round of text mining. A semantic network map was generated by extracting terms related to “hazardous accident” terms, and communities were explored to pinpoint actual injury risk issues based on the connections between terms and communities.

### Semantic network analysis

Semantic network analysis comprises nodes and links, where nodes represent terms with distinct properties, and links denote connections between terms. To establish connections or links between terms, an analysis is conducted to identify terms frequently used together or appearing simultaneously in the same document. The phi (φ) coefficient serves as a measure for identifying binary correlations between terms in a document (31). The correlation coefficient is interpreted as follows: 0.05–0.10 indicates a weak correlation, 0.10–0.15 indicates a moderate correlation, 0.15–0.25 indicates a strong correlation, and 0.25 or higher indicates a very strong correlation (32, 33). In this study, the correlation coefficient between terms was calculated using Table 2 and Equation (4) and was considered a network link to construct a semantic network map.


(4) $$\Phi =\frac{\mathrm{AD}-BC}{\sqrt{\left(A+B\right)\left(C+D\right)\left(A+C\right)\left(B+D\right)}}$$

**Table 2:** Frequencies of the two words within each line (29).

	Contains	Does not contain
Contains	A	B
Does not contain	C	D

### Community exploration

Community exploration is a process for identifying groups of nodes that interact within a network based on structural characteristics, which represents a crucial step in understanding various network structures applied across diverse fields (34). Within a community, numerous internal links foster cohesion, whereas fewer links between communities lead to separation. Modularity serves as a metric for evaluating community splits, calculated by comparing them to a random baseline rather than an absolute value. This implies that the optimal community separation corresponds to the point when modularity is maximized (35). Although various algorithms exist for community analysis, the Louvain algorithm (36), a modularity optimization method, is employed here. The Louvain algorithm initiates with each node forming its own cluster and progressively merges pairs of clusters until achieving maximal modularity (35). Its advantage lies in not needing to calculate all nodes each time, thereby reducing computation time by simplifying the modularity change equation.

## Results

### Results of term importance analysis

TF-IDF weights were computed to assess the significance of terms in paramedic evaluations of injury accidents each year, and the top 300 terms were chosen as “candidate injury keywords,” which were then sorted by the average annual TF-IDF weight. Table 3 presents a summary of the results for 20 of the leading 300 TF-IDF terms on an annualized basis.

**Table 3:** List of top 20 candidate injury keywords based on annualized TF-IDF.

	Word	2013	2014	2015	2016	2017	2018	2019	2020	2021	2022	Average
1	Snowy road	6.11	6.02	7.46	6.59	6.47	6.35	0.55	0.74	0.76	0.47	4.15
2	Escalator	7.58	7.29	6.80	6.98	1.40	1.71	1.33	1.34	1.23		3.65
3	Rice paddy	7.36	7.09	3.73	6.68	6.50	0.82	1.27	0.55	0.80	0.69	3.55
4	Pratfall	5.71	5.83	5.42	5.41	5.09	4.76	1.13	0.64	0.74	0.71	3.54
5	Thorn	3.56	6.92	6.80	6.98	2.36	3.33	1.16	2.19	1.32	0.76	3.54
6	Bathhouse	5.37	5.58	5.79	5.60	2.72	5.52	1.83	1.17	0.75	0.72	3.50
7	Object	6.38	6.63	3.13	3.15	6.17	6.06	0.98	0.84	0.91	0.73	3.50
8	Icy road	4.66	5.05	6.43	5.95	5.15	1.42	0.96	1.09	1.75	2.21	3.47
9	Insect	6.24	2.97	6.11	6.03	3.84	3.83	1.19	1.90	1.30	1.25	3.47
10	Centipede	5.88	5.35	5.29	5.28	5.16	2.52	1.70	1.64	0.86	0.94	3.46
11	Hornet	5.76	5.52	5.54	5.18	5.70	2.62	1.31	0.92	1.26	0.73	3.45
12	Bathroom	5.55	5.62	5.48	5.53	5.49	1.88	1.13	1.37	1.50	0.77	3.43
13	Wood floor	6.66	3.36	6.73	6.71	6.64	1.23	0.86	0.64	0.81	0.58	3.42
14	Bite	5.89	5.89	6.26	5.90	2.94	2.10	1.62	1.30	1.13	1.07	3.41
15	Kitchen	6.54	6.75	6.61	6.62	3.16	1.50	0.73	0.48	0.79	0.67	3.38
16	Firecracker	7.43	2.40	7.46	7.81	7.74	0.00	0.73	0.00	0.00	0.00	3.36
17	Mowing	6.24	6.63	6.06	5.43	3.01	1.93	1.08	1.48	0.76	0.85	3.35
18	Stomachache	4.72	4.69	5.04	5.21	5.44	5.50	1.08	0.57	0.63	0.56	3.34
19	Motorcycle	5.72	5.39	5.29	5.35	5.15	1.50	1.33	1.34	1.35	0.81	3.32
20	Bitten	6.38	3.37	3.44	7.16	6.70	1.14	1.63	1.53	0.80	0.94	3.31

TF, Term frequency; IDF, inverse document frequency.

The terms “snowy road,” “escalator,” “rice paddy,” “pratfall,” “thorn,” “bathhouse,” “object,” “ice road,” “insect,” and “centipede” were ranked as the most important. Examining the TF-IDF weighting results by year in Table 3, most terms, including those mentioned, have diminished in importance in recent years compared to their importance in the past. This trend is likely attributed to a decrease in the number of injury accidents in the study area over the past decade and an increase in the volume of assessment reports and the terms used. As the total number of injury accidents is applied to the total number of documents in Equation (3), a reduction in injury accidents directly leads to a decrease in IDF. Moreover, the revision of the standardized EMS guidelines for 119 paramedics has provided a more specific method of writing evaluation opinions than in the past. The quality control of 119 emergency services, based on recorded information such as EMS activity logs, has increased the volume of paramedic evaluation opinions and enhanced information delivery power. As indicated in Table 1, the number of terms recorded in the assessment findings increased over time, estimated to have reduced the TF value inversely proportional to the total number of words in the document according to Equation (2).

Among the 300 candidate injury keywords, terms with the most extensive standard deviation of TF-IDF weights across years included “firecracker,” “yawn,” “car door,” “snowy road,” and “escalator,” with “yawn” and “car door” showing particularly significant increases and decreases in TF-IDF weight across years. Terms with the highest average TF-IDF weight over the last 3 years encompassed “dialysis” (2.65), “grass door” (2.44), “company” (2.31), “fishing” (2.01), and “toenail” (1.83). The terms with the highest TF-IDF weight in 2022, the most recent year for which data are available, were “company” (5.62), “height” (3.39), “toenail” (2.76), “fall” (2.51), and “icy road” (2.21).

### Categorization and keyword selection

The average frequency, relative volatility, and average acceleration were computed for the 300 terms chosen as candidate injury keywords and converted to an ordinal scale of 1–5. As displayed in Table 4, terms with high average frequency (Point 4, Point 5) exhibit a lower tendency to be centered than terms with low average frequency (Point 1, Point 2), particularly terms within the top 20% of average frequency (Point 5), which has a standard deviation of 448.55. Conversely, for average acceleration, the standard deviations of the top 20% (point 5) and bottom 20% (point 1) terms are relatively large, indicating that certain terms have very fast acceleration or deceleration trends. As terms with average acceleration scores in the bottom 40% (point 1, point 2) have negative average acceleration, the rate of appearance of these terms is decreasing. Similar to the average frequency score, the relative volatility score shows that the top-ranked terms have a relatively large standard deviation.

**Table 4:** Summary statistics by ranking scoreband.

	Average frequency				Average acceleration				Relative volatility			
	Min.	Max.	Mean	SD	Min.	Max.	Mean	SD	Min.	Max.	Mean	SD
1-point terms (Ranking 80–100%)	5.50	29.50	20.82	6.42	−27.25	−2.13	−6.45	5.84	0.03	0.13	0.09	0.02
2-point terms (ranking 60–80%)	30.20	51.70	40.49	5.68	−2.00	−0.38	−1.17	0.48	0.13	0.22	0.17	0.03
3-point terms (ranking ~60%)	52.30	90.30	68.77	10.13	−0.25	1.13	0.38	0.39	0.23	0.41	0.31	0.05
4-point terms (ranking 20–40%)	92.70	242.30	151.72	42.42	1.25	3.38	2.17	0.61	0.41	1.02	0.62	0.18
5-point terms (ranking top 20%)	265.70	2233.50	673.08	448.55	3.50	70.88	11.98	11.08	1.12	12.68	3.03	2.22

SD, standard deviation.

Based on the ranking scale scores for average frequency, relative volatility, and average acceleration, the injury keywords were categorized into three groups (hazardous, noteworthy, and diffusion accidents), as presented in Table 5. To enhance the efficiency of the analysis, terms related to the time of day (afternoon, morning, etc.), person (daughter-in-law, acquaintance, etc.), and injury site (wrist, legs, etc.) were excluded when selecting keywords. These terms are not useful for narrowing down the scope of a wide range of injury types. However, some terms, especially those related to the time of day, may be crucial factors in understanding injury mechanisms. In such cases, these terms will be categorized as key terms with high connectivity in future semantic network analyses. This led to the classification of 25 “hazardous accidents,” 12 “noteworthy accidents,” and 28 “diffusion accidents,” as demonstrated in Table 6. The characteristics of each category of keywords can be summarized as follows.

**Table 5:** Score table of average frequency, average acceleration, and relative volatility by term category.

	Average frequency	Average acceleration	Relative volatility
Hazardous accident	4 points or more	4 points or more	4 points or more
Noteworthy accident	2 points or less	3 points or more	3 points or more
Diffusion accident	4 points or more	2 points or less	–

**Table 6:** Statistical characteristics and representative keywords by term category.

		Hazardous accident	Noteworthy accident	Diffusion accident
Number of terms (N)		25	12	28
Mean (M)	Average frequency	357.69	42.70	334.27
	Average acceleration	13.14	3.06	−6.63
	Relative volatility	2.11	0.34	1.04
Representative terms		Fall, toilet, Soju, bed, athletic, chair, icy road, etc.	Dizziness, icy road, snowy road, intravenous fluids, etc.	Task, stairs, bicycle, assault, cough, burn/scald, etc.

**Figure 2:**
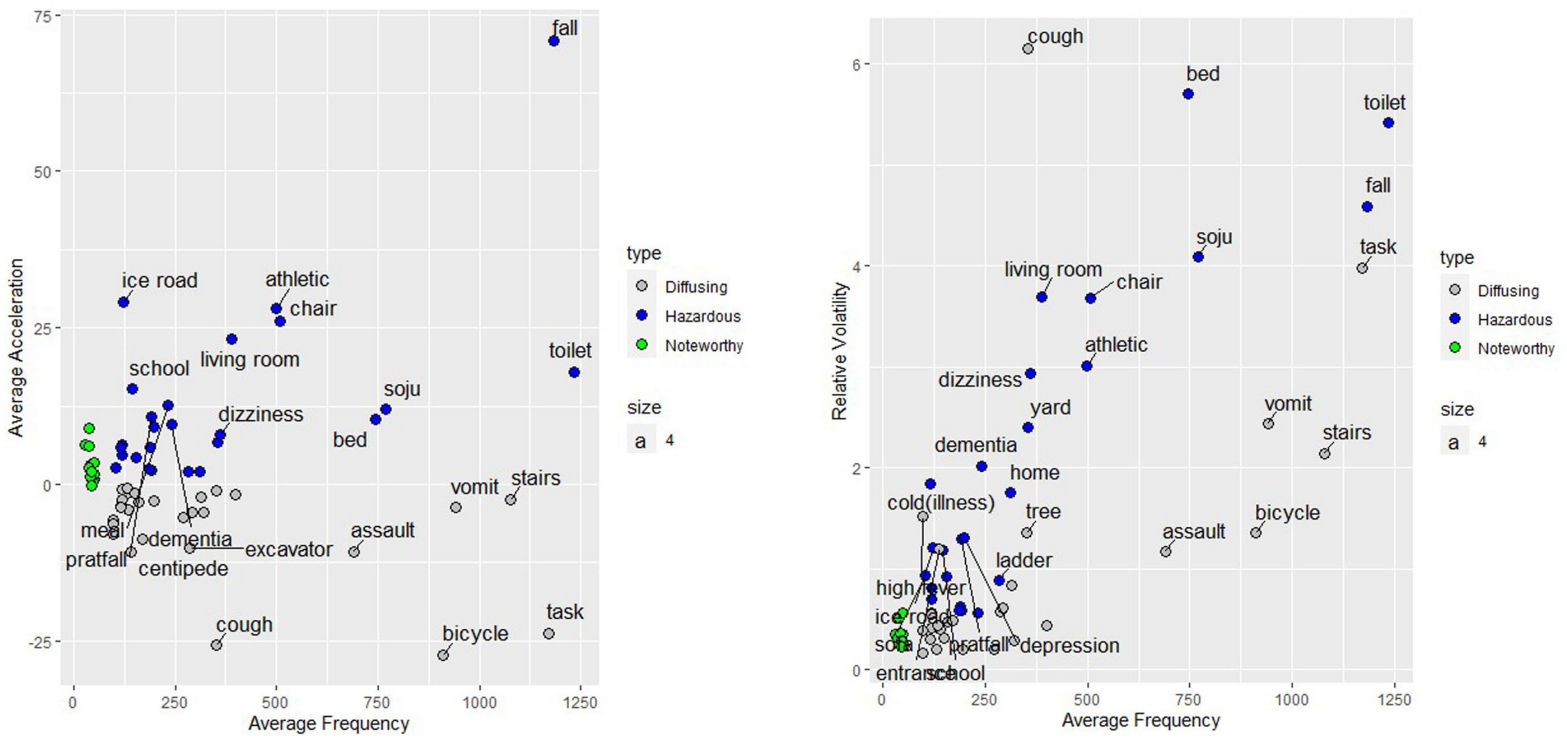
Semantic network analysis related to hazardous accident terms.

The “hazardous accident” group is defined as terms with an average frequency, average acceleration, and relative volatility score of 4 or higher, indicating very high average frequency, acceleration, and relative volatility (Figure 2). This implies that there have been numerous accidents involving the term in the last 10 years, the rate of increase is high, the volatility is high, and it is likely to increase sharply at any moment. Therefore, it can be interpreted as an accident term with the highest risk in the future. Examples include “fall,” “toilet,” “Soju,” and “athletic.” “Noteworthy accident” terms are those with an average frequency of 2 or less and an average acceleration and relative volatility of 3 or more. Accidents that are relatively infrequent but have higher than moderate acceleration and relative variability (3 or more points) include “dizziness,” “icy road,” “snowy road,” and “intravenous fluids.” These terms are characterized by an uncertain future trend of increasing or decreasing accidents, which may increase or decrease rapidly as the social environment changes. “Diffusion accident” terms are terms with an average frequency of 4 or more but an average acceleration of 2 or less. As these are terms that have a high frequency of accidents and reduced acceleration, it is likely that the risk is already recognized, and various preventive measures are in place. Examples include “task,” “stairs,” “bicycle,” “burn/scald,” and “assault.”

### Semantic analysis of hazardous accident keywords

By selecting 32,918 injury accidents over the past 10 years that were related to 25 “hazardous accident” terms with high urgency for policy introduction, data pre-processing and downsizing processes were performed (more than 10 injury accidents). The phi coefficient between the terms was calculated and applied to the links in the network. Based on this, the ego network centered on the keywords of hazardous accidents was extracted. An ego network is a subnetwork composed of selected nodes and their neighbors, called egos (35), and is often used when the number of nodes makes it difficult for researchers to capture meaningful information. In this study, 25 hazardous accident keywords were applied as egos to extract a network consisting of nodes that form direct links with the keywords and links between them. Furthermore, a community analysis was conducted to examine how the network was organized and how it functioned.

The total number of nodes in the network was 3,892, and the number of links was 97,275: thus, there were 3,867 terms with at least 10 co-occurrences with the 25 hazardous accident terms in the last 10 years. Although the scope of the network was reduced by extracting the ego network, this was still rather large (in terms of the number of nodes and links). Therefore, the correlation matrix had to be resized to reduce the number of links to make the network more readable. In this study, the threshold of the correlation coefficient, which was the basis of the correlation matrix, was set to 0.05 (32)–a threshold value that indicated weak correlation, to filter out links. The adjusted network consisted of 320 nodes and 373 links.

There were a total of 39 pairs of terms with a phi coefficient of 0.15 or higher, as shown in Table 7. In particular, the correlation coefficients of “exercise” and “sensation,” and “wheelchair” and “motorized” were over 0.5, indicating a very close correlation. “Cold,” “athletic,” and “depression” had the highest number of closely related terms (8, 6, and 5, respectively), whereas “ladder,” “self-harm,” “soccer,” and “dislocation” were analyzed as being closely related to 3 terms each.

**Table 7:** Correlation coefficients and co-occurrence frequencies for pairs of highly correlated terms.

No.	Term 1	Term 2	Coefficient	Co-occurrence N	No.	Term 1	Term 2	Coefficient	Co-occurence N
1	Athletic	Sense	0.565	2,731	21	Cold	Transfer	0.203	191
2	Wheelchair	Electric	0.544	654	22	Cold	Overseas trip	0.197	95
3	Ladder	Task	0.366	1,272	23	Dislocation	Doubt	0.194	501
4	self-harm	Wrist	0.333	1,013	24	School	Teacher	0.186	116
5	Ladder	Drop	0.304	668	25	Depression	Sleeping pill	0.180	314
6	Soccer	Game	0.290	258	26	Cold	Energy	0.179	121
7	School	Elementary	0.285	123	27	Depression	Panic	0.176	124
8	Ladder	Height	0.284	842	28	Self-harm	Fruit knife	0.174	114
9	parking lot	Underground	0.278	137	29	Soccer	Playground	0.173	187
10	Cold	Domestic	0.266	89	30	Dementia	Nursing home	0.170	292
11	Athletic	Function	0.252	417	31	Cold	Cough	0.169	264
12	Dislocation	Habit	0.241	149	32	Soccer	Player	0.169	74
13	Cold (illness)	Symptom	0.239	909	33	Cold	Finished	0.166	291
14	Dislocation	Shoulder	0.237	769	34	Athletic	Circulation	0.164	201
15	Depression	Dosing	0.228	898	35	Depression	Prescription	0.164	235
16	Meal	Lunch	0.226	255	36	Athletic	Instrument	0.160	211
17	Cold	Glove	0.211	61	37	Dizziness	Vomit	0.160	787
18	*Soju*	Half bottle	0.205	418	38	Depression	Suicide	0.154	232
19	Athletic	Ability	0.205	247	39	Self-harm	Attempt	0.150	237
20	Athletic	Pulse	0.204	823					

**Figure 3:**
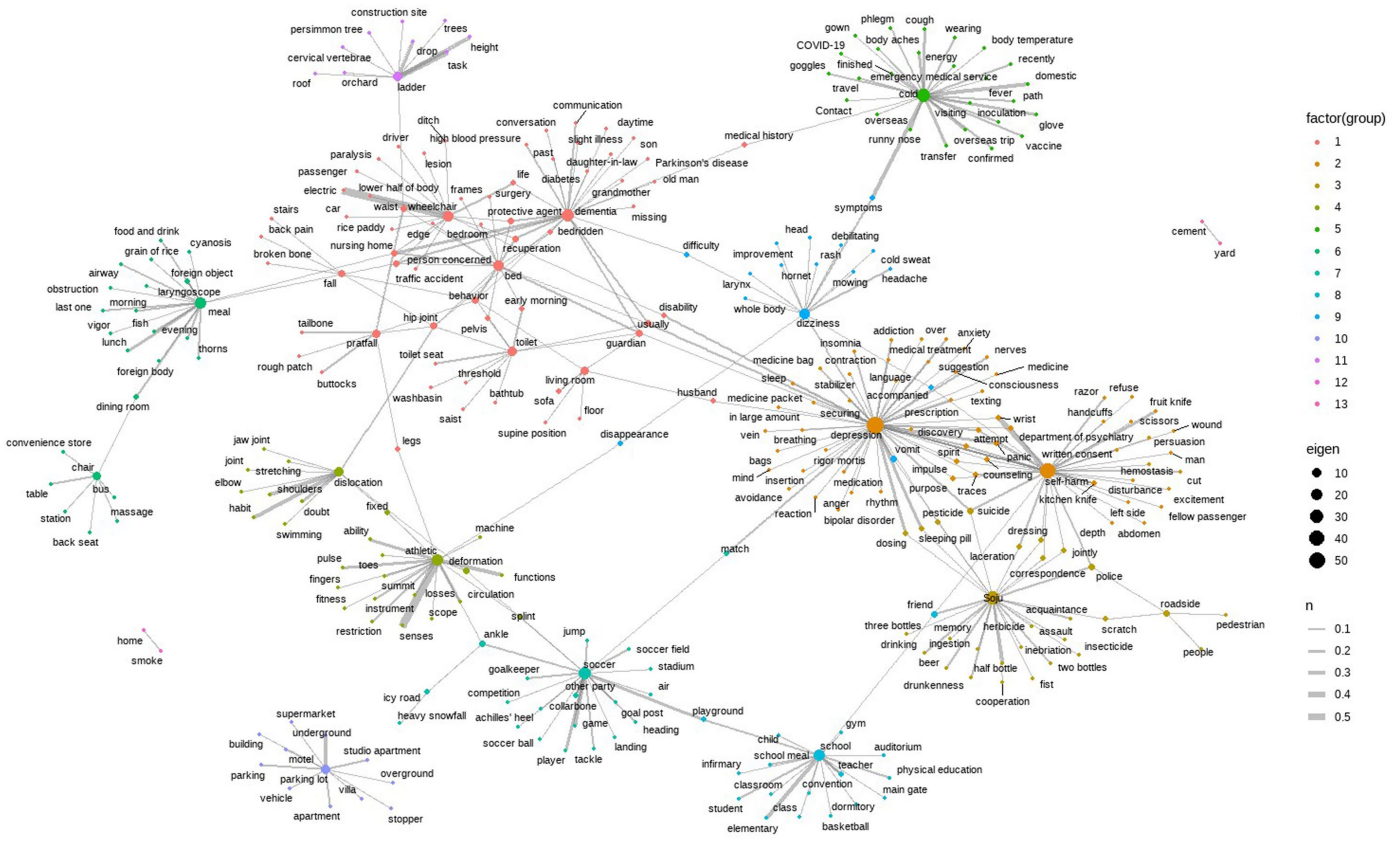
Scatterplot of statistical characteristics and accident keywords.

According to the community analysis, there were 13 communities, as shown in Table 8 and Figure 3. The hazardous accident keywords “depression” and “self-harm” have a degree of 52 and 38, respectively, making them the most connected terms in the entire network. The subnetwork of “depression” and the subnetwork of “self-harm” formed one community (C2) with “department of psychiatry,” “panic,” “impulse,” “attempt,” “counseling,” etc., and showed a highly dense network based on the high number of connections. For “self-harm,” both self-harm means (fruit knife, kitchen knife, razor, scissors) and self-harm parts (abdomen, wrist, left side) were mainly connected whereas, for “depression,” drug-related terms, such as “medicine,” “insomnia,” “medication,” and “stabilizer,” were mainly connected. Based on the types of terms and connections that make up the community, it could be inferred that depression was causing impulsive self-harm attempts in the form of drug overdoses or cuts with sharp objects.

**Table 8:** Community analysis results from the hazardous accident keyword network.

Community	Number of nodes	Nodes
C1	59	Wheelchair, dementia, toilet, bed, living room, fall, sofa, electric, nursing home, early morning, communication, toilet seat, high blood pressure, recuperation, guardian, disability, protective agent, hip joint, person concerned, etc.
C2	61	Self-harm, depression, wrist, panic, fruit knife, prescription, attempt, medicine bag, spirit, kitchen knife, cut, razor, medication, over, traces, insomnia, addiction, nerves, abdomen, wound, etc.
C3	29	Soju, roadside, dosing, half bottle, sleeping, pill, suicide, drunkenness, laceration, police, beer, two bottles, drinking, acquaintance, pedestrian, dressing, herbicide, jointly, correspondence, pesticide, scratch, etc.
C4	27	Athletic, dislocation, senses, functions, habit, shoulders, ability, pulse, doubt, circulation, instrument, restriction, summit, fitness, fixed, elbow, fingers, splint, machine, losses, etc.
C5	27	Cold, domestic, glove, transfer, overseas trip, energy, cough, finished, confirmed, phlegm, goggles, recently, path, runny nose, vaccine, wearing, inoculation, body aches, travel, contact, COVID-19, etc.
C6	24	Meal, chair, lunch, evening, food and drink, airway, foreign object, last one, table, dining room, thorns, fish, morning, grain of rice, laryngoscope, massage, obstruction, cyanosis, bus, vigor, etc.
C7	21	Soccer, icy road, game, player, soccer field, other party, heading, ankle, tackle, jump, goalkeeper, goal postmatch, air, competition, heavy snowfall, collarbone, landing, stadium, Achilles’ heel, soccer ball
C8	18	School, elementary, teacher, playground, physical, education, school meal, friend, student, gym, classroom, infirmary class, dormitory, main gate, basketball, convention, auditorium, child
C9	16	Dizziness, symptoms, vomit, headache, difficulty, head, hornet, improvement, larynx, cold sweat, rash, whole body, accompanied, disappearance, debilitating, mowing
C10	12	Parking lot, underground, apartment, parking, studio apartment, stopper, vehicle, overground, supermarket, motel, villa, building
C11	16	Ladder, pratfall, task, drop, height, waist, buttocks, tailbone, cervical, vertebrae, roof, rough, patch, construction site, legs, orchard, persimmon tree, trees
C12	2	Home, smoke
C13	2	Yard, cement

COVID-19, coronavirus disease 2019.

The subnetworks organized around the keywords “wheelchair” (degree 16), “bed” (degree 16), “toilet” (degree 11), “dementia” (degree 25), “fall” (degree 5), and “pratfall” (degree 7) were organized into one community (C1) through the terms “nursing home,” “recuperation,” “protective agent,” “bedridden,” “hip joint,” and “behavior.” “Wheelchair” was connected to terms related to traffic accidents (car, passenger, driver) and places of accidents (rice paddy, ditch, nursing home), and “dementia” formed linkages with terms related to people around (son, daughter-in-law, grandmother, old man) and underlying diseases (high blood pressure, Parkinson’s disease, diabetes). “C1” was a community dedicated to the topic of injury accidents among the older adult with low physical and mental health levels. As for “fall,” the average frequency of occurrence over 10 years was extremely high (Figure 2), while there were relatively few terms that form direct links. This means that while falls occur frequently, they occur in a variety of situations with no specific hazardous locations or factors. This community (C1) was connected to a smaller community (C11) centered around “ladder” (degree 10) through “waist.” “C11” was analyzed as comprising terms related to the location of the crash (orchard, trees, construction site, roof).

The community (C3) centered on the keyword “*Soju*” (degree 27) consisted of terms that indicated the patient’s condition due to alcohol consumption (drinking, inebriation, drunkenness), assault-related terms (fist, laceration, scratch, police), and pesticide poisoning terms (pesticide, suicide, dosing), and was highly related to “C2.” “Sleeping pill,” “dosing,” “pesticide,” and “suicide” were linked to “depression” in “C2,” indicating that depressed patients who consumed alcohol (*Soju*) often attempted suicide by taking pesticides, sleeping pills, etc. For “self-harm” in “C2,” links were formed with “dressing,” “laceration,” “police,” “correspondence,” and “jointly” in “C3.” This means that there are many cases of violent self-injurious behavior after alcohol (soju) consumption that result in a joint police response.

The subnetwork centered on the keyword “meal” (degree 19) was organized into one community (C6) with a subnetwork centered on the keyword “chair” (degree 7) and the node “dining room.” “Meal” is connected with food terms (grain of rice, fish, foreign object, thorns) and patient condition terms (airway, obstruction, cyanosis), and the community can be defined as an accident that occurs when food gets stuck in the airway during a meal.

The community (C5) organized around the keyword “cold” (degree 28) was affected by COVID-19 and consisted of terms related to prevention (glove, vaccine, goggles, COVID-19, wearing, inoculation), terms to identify the route of infection (overseas trip, visiting, overseas, contact, travel, domestic, path), and terms for cold symptoms (body aches, phlegm, runny nose, fever). Through the mediation of “symptoms,” it was connected to a community (C9) centered on “dizziness” (degree 15), but the connectivity between communities for injury causes and conditions was relatively weak. Considering the terms “mowing,” “hornet,” “vomit,” and “rash” linked to “dizziness” in “C9,” the community can be defined as various symptoms caused by wasp stings during outdoor activities such as mowing.

The subnetwork centered on the keyword “athletic” (degree 20) was connected to the subnetwork centered on “dislocation” through “fixed” and “deformation” to form one community (C4). “Athletic” was especially highly correlated with “sense” and “function” because paramedics essentially checked motor function and sensation in the event of an injury accident and included them in their assessment. “Athletic” has the meaning of physical movement, but it can also refer to activities to improve health, so careful interpretation is required. In the latter case, it forms a link with the type of sport (fitness, swimming, stretching).

The community (C8) centered on the keyword “school” (degree 18) and the community (C7) centered on the keyword “soccer” (degree 22) were connected through “playground.” “School” is connected mainly with injury location-related terms (dormitory, classroom, gym, auditorium, infirmary, main gate), and “soccer” is connected mainly with injury-inducing action terms (air, jump, landing, competition, tackle, heading).

“C12” and “C13” are analyzed as communities consisting of only two nodes and one link, where ‘home’ is connected to “smoke,” and “cement” is connected to “yard.” For both communities, the lack of components made it difficult to identify a clear sense of the meaning.

## Discussion

In this study, injury keywords were selected, classified, and defined according to categories, and the problem of injury risk was specified through semantic network analysis. Among the key findings, three injury issues that were likely to lead to an increase in risk in the future were summarized, along with their implications.

First, when analyzed based on the annualized term importance (TF-IDF) (Table 3), “snowy road” and “icy road” were found to be very important. In particular, “icy road” showed a sharp increase in the number of related injury accidents in recent years – from 32 in 2020 to 120 in 2021 and 300 in 2022. It was categorized as a “hazardous accident” term with a high average frequency, relative volatility, and average acceleration based on this time-series trend change (Figure 2). Through the linkage of “icy road” with “ankle” and “heavy snowfall” within the seventh community (C7), it can be inferred that there is a significant increase in ankle injuries due to slip-and-fall accidents on icy roads as a result of abnormal weather conditions such as heavy snowfall and cold snaps. It is predicted that extreme anomalies in average surface temperatures will increase by 32% across East Asia due to global warming, with an increase in cold snaps similar to the one experienced in East Asia in January 2016 (37). Severe cold snaps are known to cause not only direct health effects such as hypothermia and frostbite but also indirect health effects such as limb fractures due to slip-and-fall accidents on icy roads and traumatic brain injuries. According to previous studies, winter seasonal fractures, exceeding seasonal fluctuations significantly, occur on days with low temperatures and precipitation such as rain or snow (38–41). Hence, it is deemed important to emphasize the potential increase in the risk of slip-and-fall accidents on icy roads due to future extreme weather events. However, as media attention emphasizes traffic accidents, the risk of falls is often overlooked. There is a need to demonstrate the relationship between weather variables, severe weather warnings, and fracture prevalence for healthcare planning and to manage fluctuations in healthcare demand (39). Winter fractures have the characteristics of a “major accident”; however, they differ from injuries caused by major accidents in that they can be predicted and prevented. Immediate cleaning of pavements in city centers and other areas with heavy pedestrian traffic and providing the public with practical advice on how to walk more safely on slippery surfaces are essential (38).

Second, “depression” and “self-harm” formed a highly dense network based on a large number of related terms (Figure 3). Associated terms were primarily related to drugs, sharp self-injury tools, and self-injury sites, and were also highly associated with communities centered around “*Soju*,” a popular type of alcohol in South Korea. The results of multiple prior studies provide further support for the analysis of injury risk issues like this, adding to its credibility. Depression and suicide are closely related, as depression is the psychiatric diagnosis most commonly associated with suicide (42, 43). Any situation that negatively affects an individual is known to have the potential to trigger depressive symptoms and eventually lead to suicidal behavior (44). As with completed suicide, people who self-harm are likely to suffer from depression, and subsequent suicide rates are high, especially for those with persistent depression (45, 46). According to a specific study, there is a tendency for suicide rates and self-harm rates to increase when starting or discontinuing antidepressants. Therefore, it is important to exercise caution not only when initiating treatment for depression but also when discontinuing it (47). It is also known that alcohol can acutely increase the risk of self-harm through several mechanisms 48. In this study, the frequency of “depression” increased sharply to 96 in 2014, 203 in 2018, and 369 in 2022, and the frequency of “self-harm” increased proportionally to 136 in 2014, 198 in 2018, and 265 in 2022. Depression and self-harm not only form a large semantic network through multiple terms but also show a linear relationship in quantitative terms. The change in the time-series trend confirms that the problem may have a more negative social impact in the future. Based on the results of previous studies analyzing the relationship between depression and various variables such as stress, self-harm, suicide, and alcohol consumption, early management and psychological and social treatment for self-harm and suicide due to depression seem to require further reinforcement. In particular, measures will be needed to strengthen access restrictions for drug overdose and self-cutting – the methods of self-harm identified in this study. Cutting the skin with a sharp object such as a razor, glass, or knife is the most common form of self-harm (48). Although cutting-based forms of self-harm have been described since ancient times, drug overdoses have emerged after the significant growth in relatively safe pharmaceutical products (49). In the United Kingdom, the number of paracetamol overdoses has decreased significantly since legislation was amended in 1998 to require packaging units of painkillers to be below the lethal dose (50). Therefore, effective measures are needed to prevent physical access to suicidal means in South Korea. According to South Korea’s Fifth National Suicide Prevention Master Plan, released in February 2023, to reduce suicide risk factors, antiepileptic drugs, sedatives, sleeping pills, antiparkinsonian drugs, and sodium nitrite, known as suicide drugs, will be included in the online suicide risk notices for the next 5 years, and monitoring will be strengthened. In light of these findings, effectiveness is expected to increase if a specific implementation plan is prepared to limit physical accessibility in combination with cognitive access prevention, such as media measures, SNS monitoring, and blocking of harmful sites.

Finally, the community’s terminological organization of keywords such as “wheelchair,” “bed,” “toilet,” “dementia,” and “fall” confirms that older adults with low levels of physical and mental health are at an increased risk of injury accidents. The keyword to describe the injury activity in this community is “fall,” with other terms being structured around environmental and biological risk factors, which are defined as the main risk factors for falls (biological/behavioral/environmental/socioeconomic) (51). As mentioned above, “fall” has been categorized as a high-risk term due to its high frequency of occurrence, rapid rate of increase, and high volatility in the last decade (Figure 2). A fall is a prominent external cause of unintentional injury and is usually defined as an inadvertent movement to the ground, floor, or lower level (51). Globally, adults aged 65 years and older experience falls more often than younger individuals, often resulting in serious injuries and increased healthcare costs. Gait and balance disorders in older adults are one of the most common causes of falls, with a negative impact on quality of life and survival (52). In this regard, falls are considered a major public health issue, and more people will be at risk of falls with the growth of the aging population. An analysis of large cohort data found that higher age, polypharmacy, malnutrition, smoking, and alcohol use significantly increased the risk of falls, and that individuals with heart disease, hypertension, a history of falls, depression, and pain were at higher risk of falls than those without these comorbidities (53). The semantic network of this study also confirmed this trend, as terms that can infer comorbidities, such as “dementia,” “Parkinson’s disease,” “medical history,” “diabetes,” and “surgery,” were grouped into the same community as “fall”. Using two or more medications increases the risk of falls and injuries among the older adult (54), particularly antihypertensive medications, one of the factors in the Downton Fall Risk Index (DFRI), have been shown to increase the risk of serious injuries due to falls (55, 56). The issue of falls among the older adult has been recognized as an important topic from a public health perspective for the past several decades, and this study also confirmed comprehensive risks related to older adult falls through text mining. With the rapid aging of Korean society, unintentional falls among the older adult are predicted to have significant medical and economic consequences in the future. Given the geographic and ethnic limitations, studies focusing on older adults in South Korea are needed to identify demographic characteristics, comorbidities, and lifestyle factors that influence the risk of falls, which should facilitate the development of effective fall prevention strategies.

The ultimate goal of an injury prevention policy is to identify and reduce potential injury risks or issues through the analysis of current and historical conditions. These potential injury risk issues can significantly impact the magnitude and social consequences of future injuries. In this study, TF-IDF weights, commonly used in text mining research, were employed to select candidate injury keywords. Statistical analysis indicators, particularly “novelty” and “scalability,” among various features defining emerging issues, were then utilized to derive final keywords, which were subsequently categorized based on time series features. Additionally, semantic network analysis was conducted on keywords with high policy importance to explore injury risk issues. The significance of this study lies in proposing a method to promptly identify injury risk issues expected to escalate in the future by leveraging information from national EMS activities, which has been underutilized as injury surveillance data until now.

Nonetheless, this study has certain limitations. While analyzing semantic networks, a threshold for the correlation coefficient was applied to reduce the matrix for efficient analysis. However, this method could not guarantee the importance of the links. More principled approaches exist for extracting the network backbone, preserving the essential structure and overall properties of the network by identifying links responsible for a disproportionately large percentage of the connection strength of each node (35). Future work should apply these principled link filtering approaches and compare them to the results of this study.

Furthermore, as the analysis was confined to injury surveillance data from a specific region in South Korea, these data may not be considered representative of universal injury risk issues. Different cultural values, health and social services, and geographic conditions in various countries or regions are expected to result in diverse forms and causes of injury. Therefore, further research should be conducted in regions and countries with diverse injury environments to design injury-prevention policies that reflect both universal and localized characteristics.

## Data availability statement

The original contributions presented in the study are included in the article/supplementary material, further inquiries can be directed to the corresponding author.

## Author contributions

J-YW: Writing – original draft. Y-RL: Writing – original draft. M-HC: Writing – review & editing. Y-TK: Writing – review & editing. J-HL: Writing – original draft.
